# Neighborhood Risk and Hospital Use for Pediatric Asthma, Rhode Island, 2005–2014

**DOI:** 10.5888/pcd16.180490

**Published:** 2019-05-30

**Authors:** Annie Gjelsvik, Michelle L. Rogers, Aris Garro, Adam Sullivan, Daphne Koinis-Mitchell, Elizabeth L. McQuaid, Raul Smego, Patrick M. Vivier

**Affiliations:** 1 Department of Epidemiology, Brown University, Providence, Rhode Island; 2 Hassenfeld Child Health Innovation Institute, Providence, Rhode Island; 3 Department of Emergency Medicine, Brown University Warren Alpert Medical School, Providence, Rhode Island; 4 Department of Pediatrics, Brown University Warren Alpert Medical School, Providence, Rhode Island; 5 Department of Biostatistics, Brown University, Providence, Rhode Island; 6 Department of Psychiatry and Human Behavior, Brown University Warren Alpert Medical School, Providence, Rhode Island; 7 Department of Psychiatry, Rhode Island Hospital, Providence, Rhode Island; 8 Department of Health Services, Policy and Practice, Brown University, Providence, Rhode Island

## Abstract

**Introduction:**

Studies consistently show that children living in poor neighborhoods have worse asthma outcomes. The objective of our study was to assess the association between negative neighborhood factors (ie, neighborhood risk) and pediatric asthma hospital use.

**Methods:**

This retrospective study used data from children aged 2 to 17 years in a statewide (Rhode Island) hospital network administrative database linked to US Census Bureau data. We defined an asthma visit as an *International Classification of Diseases, 9th Revision, Clinical Modification* (ICD-9-CM) code of 493 in any diagnosis field. We used 8 highly correlated measures for each census-block group to construct an index of neighborhood risk. We used maps and linear regression to assess the association of neighborhood risk with average annual census-block–group rates of asthma emergency department visits and hospitalizations. We used multivariable analyses to identify child characteristics and neighborhood risk associated with an asthma revisit, accounting for the child’s sociodemographic information, season, and multiple measurements per child.

**Results:**

From 2005 through 2014, we counted 359,195 visits for 146,889 children. Of these, 12,699 children (8.6%) had one or more asthma visits. Linear regression results showed 1.18 (95% confidence interval, 1.06–1.30) more average annual emergency departments visits per 100 children and 0.41 (95% confidence interval, 0.34–0.47) more average annual hospitalizations per 100 children in neighborhoods in the highest-risk index quintile than in neighborhoods in the lowest-risk index quintile.

**Conclusion:**

Interventions to improve asthma outcomes among children should move beyond primary care or clinic settings and involve a careful evaluation of social context and environmental triggers.

**Summary**

**What is already known on this topic?**

Among children, exposure to acute and chronic stress is associated with worse asthma outcomes.

**What is added by this report?**

Our study shows that higher neighborhood risk is associated with higher odds of hospital reutilization, even after accounting for child-level factors.

**What are the implications for public health practice?**

Interventions and policies designed to address pediatric asthma should move beyond clinic settings and account for neighborhood context, with a careful evaluation of social context and environmental triggers to address the real-world challenges of managing asthma in high-risk environments.

## Introduction

Asthma is a chronic illness of the airways and is one of the most common chronic conditions of childhood (1). In 2012, asthma affected 9% (6.8 million) of children aged 0 to 17 years living in the United States (2). Although potentially preventable hospitalizations among children for all diagnoses declined from 2000 to 2007, pediatric hospital stays for asthma may have increased from 2007 to 2009 during the recession (3). In 2013, there were 571,000 emergency department (ED) visits for asthma among children aged 0 to 14 years (4).

Among children, exposure to acute and chronic stress has been associated with increased odds of an asthma diagnosis (5) and increased asthma exacerbations (6). Most studies of neighborhood context demonstrated that negative neighborhood factors — such as poverty, low high school graduation rates, and low median housing prices — are associated with higher pediatric asthma prevalence (7) and risk of adverse outcomes such as hospital use and reutilization (8–16); one study found no association (17). Prior research had several limitations. For example, most studies used census tracts to measure neighborhoods (8–15), a few used census-block groups (7,16,17), and only some accounted for clustering of hospital use by children (7,10–12,14,15,17). Block groups are the smallest unit of geography for which the US Census Bureau publishes sample data; they are less heterogeneous than census tracts (18). Without accounting for clustering of hospital use, a study may erroneously identify an association that is due to clustering as one that is due to neighborhood factors.

An ED visit by a child for an asthma exacerbation is a major disruption for the child and family and costly for society (1). Racial/ethnic minority children and poor children are more likely to visit the ED than their non-Hispanic white and nonpoor counterparts (1), and evidence indicates that individual and contextual risks are cumulative (19). Most children who visit an ED do not return to the hospital for emergency care in the subsequent year. For those who do, repeated hospital use may indicate poor asthma management, severe asthma requiring close monitoring, or both (20). Identifying predictors of repeated hospital use for children with asthma has numerous clinical implications. Such predictors may help to 1) characterize social determinants of recurrent urgent health care use for asthma and 2) identify children in need of enhanced discharge services to prevent recurrent health care use and costly hospitalization. Decreasing asthma health disparities will require action on multiple levels, including social and environmental interventions (21). It is important to increase understanding of the role of neighborhood risk in asthma hospital use and revisits.

The objective of our study was to assess the association between negative neighborhood factors (ie, neighborhood risk) and pediatric asthma hospital use, specifically, ED revisits and rehospitalizations within the subsequent year. We conducted a statewide analysis in Rhode Island, where the rate of uninsured children (2% in 2017) has been among the lowest in the country (22); this low rate minimizes the effect of financial resources on health care coverage. We formulated the following hypotheses: 1) census-block–group asthma ED visit and hospitalization rates will be higher in neighborhoods with more risk indicators, 2) children who have an index ED visit and hospitalization and who live in a high-risk neighborhood will be more likely to have a revisit within the subsequent year, and 3) these differences will persist when accounting for child-level factors.

## Methods

This retrospective study used data from a statewide hospital network administrative database in Rhode Island, the 2010–2014 American Community Survey (23), and the 2010 US Census (24). This hospital network provides approximately two-thirds of pediatric ED services and 90% of inpatient services for children living in the state (unpublished data from the 2014 Rhode Island State Emergency Department Database and 2014 Rhode Island State Inpatient Database) and includes the state’s only children’s hospital. Children aged 2 to 17 years, living in Rhode Island, with at least 1 asthma visit within this hospital network from January 1, 2005, through December 31, 2014, were identified by using the hospital network’s information systems. An asthma ED visit or hospitalization was one in which *International Classification of Diseases, 9th Revision, Clinical Modification* (ICD-9-CM), code 493 was in any diagnosis field. The child’s home address at the time of each visit was geocoded by using ArcGIS (Esri) to identify the census-block group in which the child lived (18). This study was approved by the institutional review board of the hospital network.

### Measures

**Child-level variables.** Information on the child’s age in years, sex (male, female), race/ethnicity (Hispanic, non-Hispanic black, non-Hispanic white, non-Hispanic other), and insurance coverage (private, public, self-pay/none) was recorded for each visit. We assigned season of visit according to the visit date (spring, March–May; summer, June–August; autumn, September–November; winter, December–February).

**Census-block-group–level variables.** For each census-block group, we constructed a neighborhood risk index by using 8 highly correlated census-block–group measures obtained from the 2010–2014 American Community Survey and the 2010 US Census: percentage of adults with no high school education, percentage of single-parent households, percentage of household crowding (>1 person per room), percentage of renter-occupied housing units, percentage of vacant homes (excluding vacation homes), percentage of families below 100% of the federal poverty level, percentage of nonwhite residents, and percentage of housing units built before 1950. We computed quintiles for each of the 8 measures and summed these, resulting in an index with a range of 8 to 40, with higher scores indicating greater neighborhood risk. We then computed quintiles for this index. We also dichotomized this index into high risk (at or above the 75th percentile, values of 30–40) and low risk (below the 75th percentile, values of 8–29). This dichotomization resulted in 25% (206 of 809) of census-block groups classified as high risk. These census-block groups accounted for 29% (N = 59,150) of the 2010 Census population count of children age 2 to 17 years in Rhode Island. We also calculated average annual rates of ED visits and hospitalizations by dividing the average number of visits per year by the 2010 Census estimate of children aged 2 to 17 years living in each census-block group.

**Index visit and revisits.** We focused our analysis on high rates of hospital use, specifically, revisits for asthma after an initial ED visit. We retained data on all characteristics for the initial asthma ED visit or hospitalization (index visit) during the study period. For this analysis, to allow a full 365 days for a second visit to occur for all index visits, we included only index visits occurring before 2014. Visits occurring in 2014 were included only if they were a revisit to an index visit in 2013. If information on a characteristic was missing for the index visit, we used information from the next visit with valid information. We then examined all visits occurring between 8 and 365 days after the index visit. Revisits that occurred within a few days were considered a part of the same course of illness (20). If we found one or more asthma visits within 365 days, then we coded the child as having an asthma revisit. All others were coded as either having no revisit or having a nonasthma revisit. If we found multiple asthma revisits during the period, we used the first asthma revisit in the analyses. After coding the index visit, we then processed all additional asthma visits in the same manner (ie, the first asthma revisit became the second asthma index visit for the child and a new period of 8–365 days was assessed).

### Statistical analysis

**Census-block–group level.** We first created choropleth maps to show the geographic distribution of the neighborhood risk index, the average annual rate of asthma ED visits, and the average annual rate of asthma hospitalization, by census-block group. We then used linear regression to assess the association of neighborhood risk with the average annual census-block–group rate of asthma ED visits and hospitalizations.

**Visit level.** We conducted analyses by using SAS version 9.4 (SAS Institute, Inc). We computed bivariate analyses to identify child characteristics associated with neighborhood risk at the index visit and with the occurrence of an asthma revisit. Because children can have multiple visits, we used generalized estimating equations with a repeated statement to account for the multiple measurements per child. We used an autoregressive order 1 correlation matrix to obtain dependence-corrected standard errors. The model included the child’s sex, age, race/ethnicity, and insurance coverage at the time of the index visit, the season in which the index visit occurred, and the dichotomized neighborhood risk index at the time of the index visit.

**Sensitivity analyses.** To account for children moving from one neighborhood to another between visits and potentially changing neighborhood risk level, we used a cross-classification model in the next analysis (25), grouping on the neighborhood risk at the index visit (random effect), and we included the neighborhood risk at the follow-up visit (fixed effect). Because this model required 2 visits to assess potential moves, this analytic sample included only children with at least 2 visits. We also assessed whether results were sensitive to a stricter definition of asthma, because there is no gold-standard definition of asthma using ED or inpatient data (26). In the main analyses we defined an asthma ED visit or hospitalization as ICD-9-CM code 493 in any diagnosis field, and in sensitivity analyses we counted only ED visits or hospitalizations with ICD-9-CM code 493 in the primary (first) diagnosis field as an asthma visit.

Because hospital network coverage for ED visits is lower than coverage for hospitalizations, we also conducted analyses that excluded neighborhoods that were farthest from a network hospital. The results of these analyses were the same as those of the whole state; we therefore tabulated statewide results only.

## Results

From 2005 through 2014, we counted 319,320 ED visits and 39,875 hospitalizations for 146,889 children aged 2 to 17 years in Rhode Island. Of these children, 12,699 (8.6%) had one or more asthma ED visits or hospitalizations (number of visits = 23,187). About 53% of visits were among children living in high-risk census-block groups.

**Figure 1:**
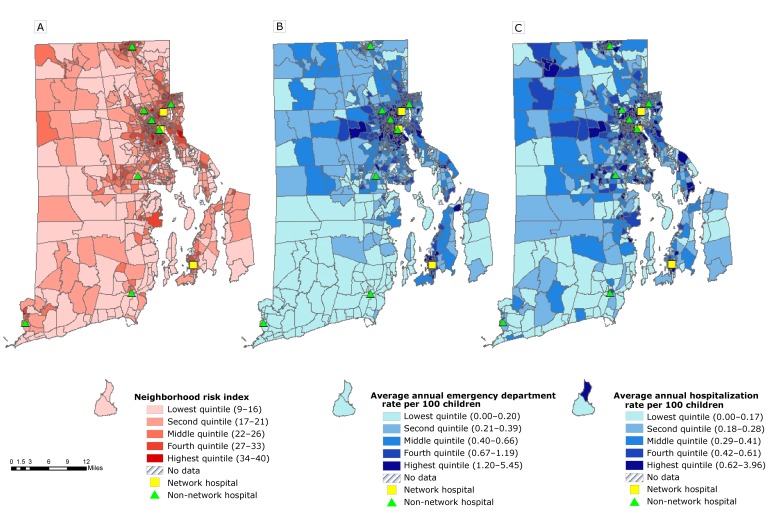
Distribution of quintiles of 3 measures used to assess the association between neighborhood risk and pediatric asthma hospital use among children aged 2 to 17 years (number of asthma emergency department [ED] visits or hospitalizations = 23,187), Rhode Island. A, Neighborhood risk index; the higher the index, the higher the prevalence of adverse socioeconomic and health-related factors, 2010–2014. B, Average annual emergency department visit rate per 100 children, 2005–2014. C, Average annual hospitalization rate per 100 children, 2005–2014. Data on neighborhood risk were collected from the 2010–2014 American Community Survey and the 2010 US Census. Data on emergency department visits and hospitalization were collected from a statewide hospital network administrative database, 2005–2014.

**Census-block–group level.** The average annual count and rate per 100 children of pediatric asthma hospital use varied across census-block groups. For ED visits, the average annual count ranged from 0 to 161 (mean 18.3, median 11.0); the average annual rate per 100 children ranged from 0.0 to 5.5 (mean, 0.7; median, 0.5). For hospitalizations, the average annual count ranged from 0 to 134 (mean, 10.3; median, 8.0); the average annual rate per 100 children ranged from 0.0 to 4.0 (mean, 0.4; median, 0.3). Linear regression results showed 1.18 (95% confidence interval [CI], 1.06–1.30) more average annual emergency departments visits per 100 children and 0.41 (95% CI, 0.34–0.47) more average annual hospitalizations per 100 children in neighborhoods in the highest-risk index quintile than in neighborhoods in the lowest-risk index quintile. The highest-risk neighborhoods were concentrated in urban areas of Rhode Island, and distribution of neighborhoods with higher rates of ED use and hospitalizations was consistent with the distribution of higher-risk neighborhoods (Figure 1).

**Figure 2:**
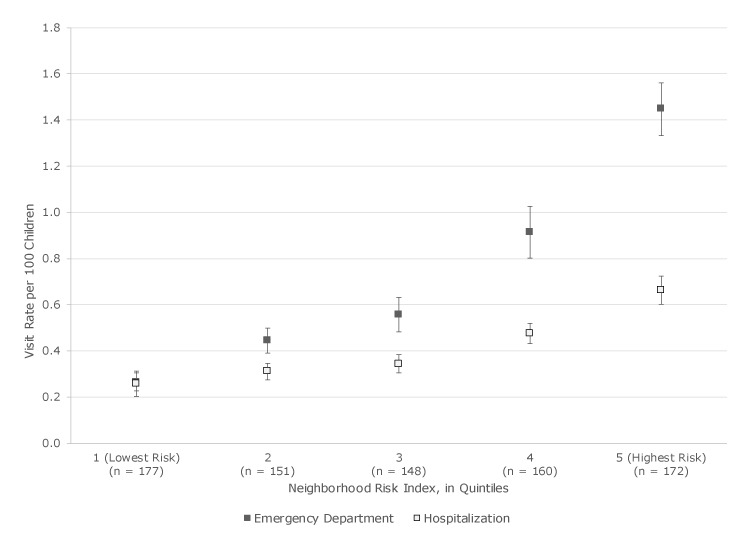
Pediatric asthma emergency department and hospitalization rates per 100 children by quintile of neighborhood risk among children aged 2 to 17 years, Rhode Island, 2005–2014. Data on neighborhood risk were collected from the 2010–2014 American Community Survey and the 2010 US Census. Data on emergency department visits and hospitalization were collected from a statewide hospital network administrative database. n’s along the *x*-axis indicate the number of census block groups. Error bars indicate standard error.

Although the average annual rate per 100 children of both pediatric asthma ED visits and hospitalization increased as neighborhood risk quintile increased, the increase was greater for ED visits than for hospitalizations. In the lowest-risk neighborhoods, the ED visit rate (0.27 per 100 children) and the hospitalization rate (0.26 per 100 children) were similar (Figure 2). In the highest-risk neighborhoods, the ED visit rate was 1.45 per 100 children and the hospitalization rate was 0.66 per 100 children.

**Table:** 

Quintile of Neighborhood Risk	No. of Census Block Groups	Visit Rate per 100 Children	Standard Error
Emergency department			
1 (Lowest risk)	177	0.27	0.02
2	151	0.44	0.03
3	148	0.56	0.04
4	160	0.91	0.06
5 (Highest risk)	172	1.45	0.06
Hospitalization			
1 (Lowest risk)	177	0.26	0.03
2	151	0.31	0.02
3	148	0.34	0.02
4	160	0.48	0.02
5 (Highest risk)	172	0.66	0.03

### Visit-level analyses clustered on child

We counted 11,547 children with an index visit occurring during the period from January 1, 2005, through December 31, 2013. After excluding visits that occurred within 7 days of the index visit (n = 860), we counted 19,700 index visits. Of these visits, 28.9% (n = 5,703) had an asthma revisit between 8 and 365 days after the index visit. Compared to children with no asthma revisit, children with an asthma revisit were younger, more likely to have public insurance, be Hispanic or non-Hispanic black, and live in a high-risk neighborhood (Table 1). Hispanic children (adjusted odds ratio [OR] = 1.24; 95% CI, 1.03–1.50) and non-Hispanic black children (adjusted OR = 1.26; 95% CI, 1.02–1.56) had significantly higher odds than non-Hispanic white children of a revisit (Table 2), and children living in high-risk neighborhoods (adjusted OR = 1.22; 95% CI, 1.00–1.48) had significantly higher odds than children living in a low-risk neighborhood. We found no significant differences by age, sex, insurance coverage, or season.

**Table 1:** Distribution of Child, Visit, and Neighborhood Characteristics for Pediatric Asthma Emergency Department Visits and Hospitalizations (N = 19,700) by Children Aged 2–17 Years in Rhode Island, 2005–2014^a^

Characteristics	No Asthma Revisit (n = 13,997)	Asthma Revisit (n = 5,703)	*P* Value
**Age, mean (SD), y**	8.1 (4.8)	7.4 (4.6)	<.001
**Sex**			
Male	59.4	59.2	.82
Female	40.6	40.8	
**Insurance coverage**			
Public	58.7	68.7	<.001
Private	37.5	27.7	
None/self-pay	3.8	3.6	
**Season**			
Winter	25.5	24.1	.18
Spring	26.5	27.3	
Summer	15.6	15.5	
Autumn	32.4	33.1	
**Race/ethnicity**			
Hispanic	31.4	38.0	<.001
Non-Hispanic black	15.3	21.5	
Non-Hispanic white	48.5	35.8	
Non-Hispanic other	4.8	4.8	
**Neighborhood risk index^b^**			
Low (<75th census-block–group percentile)	50.0	40.4	<.001
High (≥75th census-block–group percentile)	50.0	59.6	

a All values are percentages, unless otherwise indicated. Data on emergency department visits and hospitalization were collected from a statewide hospital network administrative database. We counted 11,547 children with an index visit occurring during the period from January 1, 2005, through December 31, 2013. After excluding visits that occurred within 7 days of the index visit (n = 860), we counted 19,700 index visits.

b Derived by using 8 measures from the 2010–2014 American Community Survey and 2010 US Census: percentage of adults with no high school education, percentage of single-parent households, percentage of household crowding (>1 person per room), percentage of renter-occupied housing units, percentage of vacant homes (excluding vacation homes), percentage of families below 100% of the federal poverty level, percentage of nonwhite residents, and percentage of housing units built before 1950. The index ranged in value from 8 to 40; high risk defined as an index of 30–40; low risk, 8–29.

**Table 2:** Child-Clustered Adjusted Regression Results for Pediatric Asthma Emergency Department Visits and Hospitalizations (N = 19,700) by Children Aged 2–17 Years in Rhode Island, 2005–2014^a^

Effect	Adjusted Odds Ratio (95% Confidence Interval)	*P* Value
**Age**	0.99 (0.98–1.01)	31
**Sex**		
Male	1 [Reference]	
Female	1.02 (0.93–1.11)	.72
**Insurance coverage**		
Private	1 [Reference]	
Public	1.20 (1.00–1.44)	.06
None/self-pay	1.31 (0.90–1.89)	.16
**Season**		
Winter	0.95 (0.79–1.15)	.60
Spring	1.02 (0.86–1.22)	.81
Summer	1.04 (0.84–1.29)	.71
Autumn	1 [Reference]	
**Race/ethnicity**		
Hispanic	1.24 (1.03–1.50)	.02
Non-Hispanic black	1.26 (1.02–1.56)	.04
Non-Hispanic white	1 [Reference]	
Non-Hispanic other	1.06 (0.75–1.50)	.74
**Neighborhood risk index^b^**		
Low (<75th percentile)	1 [Reference]	
High (≥75th percentile)	1.22 (1.00–1.48)	.04

a Data on emergency department visits and hospitalization were collected from a statewide hospital network administrative database. We counted 11,547 children with an index visit occurring during the period from January 1, 2005, through December 31, 2013. After excluding visits that occurred within 7 days of the index visit (n = 860), we counted 19,700 index visits. Multivariable model controlled for child and neighborhood characteristics.

b Derived by using 8 measures from the 2010–2014 American Community Survey and 2010 US Census: percentage of adults with no high school education, percentage of single-parent households, percentage of household crowding (>1 person per room), percentage of renter-occupied housing units, percentage of vacant homes (excluding vacation homes), percentage of families below 100% of the federal poverty level, percentage of nonwhite residents, and percentage of housing units built before 1950. The index ranged in value from 8 to 40; high risk defined as an index of 30–40; low risk, 8–29.

The cross-classification model, which accounted for children moving from one neighborhood to another, showed that children living in high-risk neighborhoods did not have significantly higher odds of a revisit than children living in low-risk neighborhoods (adjusted OR = 1.14; 95% CI, 0.98–1.33) (Table 3). We found significantly lower odds of a revisit among older children than younger children (adjusted OR = 0.92; 95% CI, 0.91–0.93) and index visits that occurred in the summer than in the autumn (adjusted OR = 0.77; 95% CI, 0.69–0.86). We found significantly higher odds of a revisit among girls than among boys (adjusted OR = 1.15; 95% CI, 1.06–1.25), among children with public insurance than among children with private insurance (adjusted OR = 1.10; 95% CI, 1.00–1.21), and among children who were not non-Hispanic white than among children who were (Hispanic, adjusted OR = 1.13, 95% CI, 1.02–1.27; non-Hispanic black, adjusted OR = 1.43; 95% CI, 1.26–1.62; non-Hispanic other, adjusted OR = 1.30; 95% CI, 1.07–1.58). In the sensitivity analysis in which we counted only ED visits or hospitalizations with ICD-9-CM code 493 in the primary (first) diagnosis field as an asthma visit, the adjusted odds for neighborhood risk were similar to the odds produced in the main analysis. We found no significant results in the model accounting for clustering for children in high-risk neighborhoods, compared with children in low-risk neighborhoods (adjusted OR = 1.27; 95% CI, 0.95–1.70), or the model accounting for children moving (adjusted OR = 1.02; 95% CI, 0.83–1.26) for children in high-risk neighborhoods, compared with children in low risk neighborhoods.

**Table 3:** Adjusted Cross-Classified Random-Effects Model, Clustered on Child and Neighborhood Risk, for Study on Pediatric Asthma Emergency Department Visits and Hospitalizations by Children Aged 2–17 Years in Rhode Island, 2005–2014^a^

Effect	Adjusted Odds Ratio (95% Confidence Interval)	*P* Value
**Age**	0.92 (0.91–0.93)	<.001
**Sex**		
Male	1 [Reference]	
Female	1.15 (1.06–1.25)	.001
**Insurance coverage**		
Private	1 [Reference]	
Public	1.10 (1.00–1.21)	.049
None/self-pay	0.94 (0.76–1.17)	.57
**Season**		
Winter	0.97 (0.88–1.06)	.49
Spring	1.01 (0.92–1.10)	.88
Summer	0.77 (0.69–0.86)	<.001
Autumn	1 [Reference]	
**Race/ethnicity**		
Hispanic	1.13 (1.02–1.27)	.02
Non-Hispanic black	1.43 (1.26–1.62)	<.001
Non-Hispanic white	1 [Reference]	
Non-Hispanic other	1.30 (1.07–1.58)	.008
**Neighborhood risk index^b^**		
Low (<75th percentile)	1 [Reference]	
High (≥75th percentile)	1.14 (0.98–1.33)	.08

a Data on emergency department visits and hospitalization were collected from a statewide hospital network administrative database. We counted 11,547 children with an index visit occurring during the period from January 1, 2005, through December 31, 2013. After excluding visits that occurred within 7 days of the index visit (n = 860), we counted 19,700 index visits. This cross-clarification model accounted for children who moved from their neighborhoods; after excluding these children, the number of visits was 15,156.

b Derived by using 8 measures from the 2010–2014 American Community Survey and 2010 US Census: percentage of adults with no high school education, percentage of single-parent households, percentage of household crowding (>1 person per room), percentage of renter-occupied housing units, percentage of vacant homes (excluding vacation homes), percentage of families below 100% of the federal poverty level, percentage of nonwhite residents, and percentage of housing units built before 1950. The index ranged in value from 8 to 40; high risk defined as an index of 30–40; low risk, 8–29.

## Discussion

Our study demonstrated that increased neighborhood risks contribute to pediatric ED use and hospitalizations for asthma, confirming what has been documented in previous studies (8–16). Our results add to the growing body of evidence that diverse neighborhood factors (eg, crime rates [8], housing code violation density [9], pharmacy access [11], access to primary and specialty care [13], composites of variables specified by the US Census Bureau and the American Community Survey [10,12,14–16]) in various US cities, counties, and states affect pediatric outcomes such as ED visits and hospitalizations. Regardless of the components used to measure neighborhood risk or the level of geography, studies consistently show that children living in worse neighborhoods have higher risks of ED visits and hospitalizations.

In our study, higher levels of neighborhood risk were more strongly associated with pediatric asthma ED visits than with hospitalization rates. The risk for ED visits and hospitalizations was essentially the same in the lowest-risk neighborhoods, but the difference between the 2 types of visits was wide in the highest-risk neighborhoods. This pattern is notable because ED visits are expected to be highly correlated with hospitalizations (the former frequently leading to the latter). This discrepant pattern suggests that other factors may be driving recurrent ED visits in our group of patients, such as limited skills for acute disease management and reliance on the ED for ongoing asthma care. The discrepant pattern could also suggest that proximity to the ED is a factor in rates of ED use. In our study, children living in high-risk neighborhoods had an average distance to a network hospital of 3.5 miles, whereas children living in low-risk neighborhoods had an average distance of 8.6 miles.

Another finding was that higher neighborhood risk was associated with higher odds of a revisit. This association persisted even after accounting for child-level factors. One possible explanation is that children from high-risk neighborhoods may be more likely to return to chaotic and stressful home situations and/or poor housing, where amelioration of asthma triggers is challenging. For instance, it may be more difficult to avoid environmental tobacco smoke (27) or to actively manage asthma triggers such as dust mites and pest problems in publicly financed housing than in a private home (28).

To assess the association between neighborhood risk and the odds of revisit when a child moved from one neighborhood to another between visits (and possibly changing neighborhood risk level), we conducted sensitivity analyses in which we limited the analytic sample to visits among children who had at least one revisit (asthma-related or other). When the analytic sample was limited in this way, the adjusted regression results were similar. Although few children changed neighborhood risk level from index visit to revisit (4% moved from a high-risk neighborhood to a low-risk neighborhood, 3% moved from low-risk to high-risk), when cross-classification was accounted for in regression modeling, the neighborhood risk level of the index visit was no longer significant. Reasons for this could be that the decreased sample size resulted in less power or that residential mobility itself is contributing to stress for the child and family and interruptions in care for chronic illness (29). Similarly, when the definition of an asthma visit was limited to primary diagnosis only, the adjusted odds ratios were similar but not significant. One possible reason for this could be decreased sample size (23,187 visits with ICD-9-CM as any listed diagnosis versus 13,373 visits with ICD-9-CM as primary diagnosis). It is also possible that the broader definition (any listed diagnosis) identified some visits that were not caused by asthma, even though they were visits by children with asthma (30). We found that only 37% of visits with asthma as the secondary diagnosis had respiratory illness as the primary diagnosis. The top 5 diagnosis categories, representing 76% of visits with a secondary diagnosis of asthma, were respiratory disorders (37.0%); signs/symptoms/ill-defined conditions (16.1%); injury and poisoning (8.5%); digestive disorders (7.2%); and infectious and parasitic diseases (7.2%).

Our study had several limitations. One, we obtained data on race/ethnicity from medical records that may not be reliable for these data (31). Two, the neighborhood risk index measured cumulative risk and not individual neighborhood risks. Measurement of cumulative risk was necessary because neighborhood risks are highly correlated, but it did not permit us to disentangle how each risk factor contributes to pediatric asthma hospital revisits. Three, our data were obtained from one hospital network and excluded children with out-of-network encounters. Thus, asthma-related encounters, especially ED visits, may have been underestimated. However, the hospital network represents approximately two-thirds of all pediatric ED visits and 90% of pediatric admissions and includes the only children’s hospital in the state.

Our findings provide additional evidence that interventions and policies designed to address pediatric asthma need to account for neighborhood context (9,32). Interventions that move beyond primary care or clinic setting are required. A careful evaluation of social context (family strengths and supports, financial challenges) and environmental triggers (type of housing, exposure in home and school settings) is needed. Interventions need to address the real-world challenges of managing asthma in high-risk environments. For instance, in Rhode Island, the Rhode Island Asthma Integrated Response Program (RI-AIR) is implementing a comprehensive system for screening and intervention for pediatric asthma that includes school-based education, intensive home-based interventions, and coordination among parents, school nurses, and health care providers for children whose asthma is not well controlled. A health education intervention for parents that does not account for certain factors — whether families are living in older housing stock with mold or multi-unit dwellings with inadequate ventilation or whether children are chronically exposed to triggers in older school buildings — will be less effective than a health intervention that does.
